# Alzheimer's Aβ Peptides with Disease-Associated N-Terminal Modifications: Influence of Isomerisation, Truncation and Mutation on Cu^2+^ Coordination

**DOI:** 10.1371/journal.pone.0015875

**Published:** 2010-12-30

**Authors:** Simon C. Drew, Colin L. Masters, Kevin J. Barnham

**Affiliations:** 1 Department of Pathology, The University of Melbourne, Melbourne, Victoria, Australia; 2 Neuroproteomics Platform, National Neuroscience Facility, The Bio21 Molecular Science and Biotechnology Institute, The University of Melbourne, Melbourne, Victoria, Australia; 3 Mental Health Research Institute, The University of Melbourne, Melbourne, Victoria, Australia; 4 School of Physics, Monash University, Melbourne, Victoria, Australia; 5 Centre for Neuroscience, The University of Melbourne, Melbourne, Victoria, Australia; City of Hope National Medical Center and Beckman Research Institute, United States of America

## Abstract

**Background:**

The amyloid-β (Aβ) peptide is the primary component of the extracellular senile plaques characteristic of Alzheimer's disease (AD). The metals hypothesis implicates redox-active copper ions in the pathogenesis of AD and the Cu^2+^ coordination of various Aβ peptides has been widely studied. A number of disease-associated modifications involving the first 3 residues are known, including isomerisation, mutation, truncation and cyclisation, but are yet to be characterised in detail. In particular, Aβ in plaques contain a significant amount of truncated pyroglutamate species, which appear to correlate with disease progression.

**Methodology/Principal Findings:**

We previously characterised three Cu^2+^/Aβ1–16 coordination modes in the physiological pH range that involve the first two residues. Based upon our finding that the carbonyl of Ala2 is a Cu^2+^ ligand, here we speculate on a hypothetical Cu^2+^-mediated intramolecular cleavage mechanism as a source of truncations beginning at residue 3. Using EPR spectroscopy and site-specific isotopic labelling, we have also examined four Aβ peptides with biologically relevant N-terminal modifications, Aβ1[isoAsp]–16, Aβ1–16(A2V), Aβ3–16 and Aβ3[pE]–16. The recessive A2V mutation preserved the first coordination sphere of Cu^2+^/Aβ, but altered the outer coordination sphere. Isomerisation of Asp1 produced a single dominant species involving a stable 5-membered Cu^2+^ chelate at the amino terminus. The Aβ3–16 and Aβ3[pE]–16 peptides both exhibited an equilibrium between two Cu^2+^ coordination modes between pH 6–9 with nominally the same first coordination sphere, but with a dramatically different pH dependence arising from differences in H-bonding interactions at the N-terminus.

**Conclusions/Significance:**

N-terminal modifications significantly influence the Cu^2+^ coordination of Aβ, which may be critical for alterations in aggregation propensity, redox-activity, resistance to degradation and the generation of the Aβ3–× (× = 40/42) precursor of disease-associated Aβ3[pE]–x species.

## Introduction

Alzheimer's disease (AD) is a neurodegenerative disorder characterised by progressive cognitive and memory impairment [1]. Amyloid plaques, comprising of extracellular cerebral deposits of insoluble Aβ, are the pathological hallmark of AD [1], [2]. Within these plaques, copper is found in high concentrations [2], [3] and growing evidence suggests that copper ions play an important role in the pathogenesis of AD by inducing protein misfolding and generating reactive oxygen species [4], [5], [6], [7], [8], [9]. It is generally accepted that soluble, low molecular-weight oligomers are responsible for the neurotoxic effects of Aβ [10] and although full consensus is still lacking, copper clearly influences the oligomerisation pathway of Aβ [9], [11]–[14].

The Cu^2+^ coordination of Aβ1–x peptides (× = 16, 28, 40, 42) is now well characterised, with the Aβ1–16 fragment containing all residues essential for its highest affinity coordination. Using electron paramagnetic resonance (EPR) spectroscopy and site specific ^17^O, ^15^N and ^13^C labelling, we recently introduced a refined model of Cu^2+^/Aβ interactions. Between pH 6–7, two dominant coordination modes are in equilibrium (components Ia and Ib), with a 5-membered chelate being formed between Cu^2+^, the amino nitrogen and (in at least one of the components) the backbone carbonyl of Asp1, together with nitrogen coordination by His6 and His13 (Ia) or His14 (Ib) [15], [16]. Using a similar approach, these findings have recently been reproduced [17]. At pH>7, an additional coordination mode (component II) is also populated and in equilibrium with component I species. Although the precise ligand sphere of component II remains contentious [15]–[17], we have used ^15^N- and ^13^C-labelling to identify the coordination of the carbonyl of Ala2, while site specific ^15^N-labelling and multifrequency CW-EPR simulations supported simultaneous coordination of His6, His13 and His14 in a {CO^A2^, N_Im_^H6^, N_Im_^H13^, N_Im_^H14^} coordination sphere [15].

In contrast to Aβ1–x, little has been reported about the Cu^2+^ coordination of Aβ with N-terminal truncations or modifications, yet the deposition of extracellular Aβ *in vivo* is accompanied by a large degree of amino-terminal heterogeneity. Early studies of the plaque core of AD patients identified a significant proportion of Aβ peptides with ragged N-termini [18], [19]. Of these, truncated pyroglutamate forms, particularly Aβ3[pE]–× (× = 40/42), are now believed to constitute a major component of amyloid found in senile plaques of AD patients [20]–[25] and of the amyloid detected by positron emission tomographic (PET) imaging [26]. Aβ3[pE]–40 also correlates with the extent of Aβ deposition in cerebral blood vessels [24]. The accumulation of Aβ3[pE]–x in AD is consistent with a resistance to degradation by aminopeptidases, a property displayed by a range of proteins and peptides with an amino terminal pGlu residue [27]. In both neuronal and glial cell cultures [28], [29], Aβ3[pE]–40 induces significantly more cell loss than Aβ1–40 and Aβ1–42 while intracerebroventricular injection of soluble Aβ3[pE]–42 or Aβl–42 in wild type (wt) mice leads to reduced cognitive performance and induces neuronal apoptosis *in vitro* [28]. In an APP/PS1KI mouse model of AD, a continuous rise in Aβ3[pE]–x plaque load and a concomitant decrease in Aβ1–x was observed with increasing age, suggesting that Aβ1–x peptides are N-truncated as disease progresses [30]. *In vitro*, Aβ3–x and Aβ3[E]–× (× = 40,42) aggregate to form fibrillar structures more rapidly than Aβ1–x and accelerate Aβ1–x fibril formation [31], [32], with Aβ3[pE]–40 displaying faster aggregation compared with Aβ3–40 [32]. Notably, Aβ3–42 seeding of Aβ1–40 fibril formation at pH 7.4 is greatly enhanced in the presence of substoichiometric Cu^2+^ [31]. We have demonstrated that the first two residues of Aβ1–x are both directly involved in Cu^2+^ coordination of Aβ1–16 [15], [16]; however, the coordination of the more toxic Aβ3[pE]–x species and its Aβ3–x precursor have not been investigated in detail. Furthermore, the mechanism of N-terminal truncation remains unknown.

In addition to Aβ42 and Aβ3[pE]–42, the amyloid cores from AD brain tissue contain other post-translational modifications including isomerised and racemised Aβ, in particular Aβ1[isoAsp]–42 and Aβ1[D-Asp]–42 [22], [33]. Isoaspartate (isoAsp) formation is also associated with impaired protein function and enhanced isomerisation affects both Aβ and the tau protein in AD [34]. It can be generated spontaneously from Asp and Asn residues via formation and subsequent hydrolysis of a cyclic L-succinimidyl intermediate [35] and has been shown to form upon ageing of Aβ1–16 *in vitro* [36]. Although the physiological consequences of isomerisation remain unclear, it results in an increased tendency of Aβ to form β-sheet *in vitro* [37] and was proposed to enhance the stability of Aβ deposits in AD brain tissue [33]. More recently, the Zn^2+^ coordination of Aβ1–16[isoAsp7] was studied by NMR and shown to directly coordinate via isoAsp7 (whereas Asp7 of the native Aβ1–16 does not) [36], and was subsequently shown to induce oligomerisation of Aβ1–16[isoAsp7] [38]. The latter property was proposed to be potentially relevant to the D7N Tottori–Japan mutation, in light of the greater susceptibility of asparagine to spontaneous conversion into isoAsp [38]. In contrast to isomerisation of Asp7, there are no reported studies describing the properties of Aβ1[isoAsp]–x. Given the importance of Asp1 to the Cu^2+^ coordination of the native peptide, it is of interest to characterise this species.

Tagliavini and co-workers recently identified a new recessive A673V mutation in the amyloid precursor protein that generates an Aβ(A2V) peptide [39], which appears to be associated with disease only in the homozygous carriers. *In vitro* studies of synthetic peptides demonstrated enhanced fibril formation of Aβ(A2V) in isolation, but co-incubation of wt Aβ1–40 with Aβ1–40(A2V) or even Aβ1–6(A2V) inhibited amyloid formation of the native peptide. Moreover, the viability of cultured human neuroblastoma cells was significantly reduced by Aβ1–42(A2V) compared with wt Aβ1–42. This anti-amyloidogenic effect *in vitro* was suggested to be responsible for the autosomal negative pattern of inheritance [39]. Since trace copper does not appear to have been accounted for in the above study, and the carbonyl of Ala2 in wt Aβ1–x coordinates Cu^2+^ [16], it is natural to ask how this coordination might be modified in this rare familial form of AD.

It is clear that the N-terminal modifications of Aβ, especially pGlu forms beginning at residue 3, are strongly associated with disease. Since Cu^2+^ coordination modulates peptide aggregation and toxicity of Aβ1–x [40], and this coordination involves the first two residues, we have synthesised four N-terminally modified Aβ peptides Aβ1[isoAsp]–16, Aβ1–16(A2V), Aβ3–16 and Aβ3[pE]–16, and characterised their pH-dependent Cu^2+^ coordination using EPR spectroscopy. The potential physiological consequences of the changes observed with respect to the Cu^2+^ coordination of the wt peptide are discussed and a Cu^2+^-dependent mechanism of N-terminal truncation is also hypothesised.

## Materials and Methods

### Peptide synthesis

Table 1 lists the peptides synthesised for this study. Fmoc-L-^15^N-Val-OH (^15^N, 98%) and Fmoc-L-^13^C(1)-Val-OH (^13^C(1), 99%) were purchased from Cambridge Isotope Laboratories. Fmoc-L-^13^C(1)-Asp-OH (^13^C(1), 99%), was purchased from Sigma Aldrich. Solid phase peptide synthesis was carried out in the Peptide Technology Facility of the Bio21 Molecular Science and Biotechnology Institute, The University of Melbourne, using standard protocols with HOBt/DIC as coupling reagents. Unlabelled Aβ1–16 (DAEFRHDSGYEVHHQK-OH), Aβ3–16 (EFRHDSGYEVHHQK-OH) and Aβ3[pE]–16 ([pE]FRHDSGYEVHHQK-OH) (using L-Pyroglutamic acid for the final coupling) were synthesised by solid-phase peptide synthesis on Fmoc-L-Lys(Boc)-PEG-PS resin (Applied Biosystems) using a CEM Liberty microwave peptide synthesiser. Aβ1–16(^13^C(1)-Asp1), Aβ1–16(^13^C(1)-isoAsp1), Aβ1–16(A2V,^15^N-Val2), and Aβ1–16(A2V,^13^C(1)-Val2) were similarly synthesised using the CEM Liberty microwave peptide synthesiser, except that the appropriate labelled Fmoc amino acid was manually coupled. Peptides were purified by reverse-phase HPLC. To generate Aβ1–16(^13^C(1)-isoAsp1), the N-terminal Fmoc-L-^13^C(1)-Asp-OH was coupled without any protection of the C(4)OO^−^ group. This produced both α (Asp1) and β (isoAsp1) isomers, which were then separated by RP-HPLC and their identity confirmed by Edman degradation. Using the final RP-HPLC trace, final peptide purity was determined to be > 96% for Aβ1–16(^13^C(1)-isoAsp1), > 98% for Aβ3–16, > 99% for Aβ3[pE]–16, > 92% for Aβ1–16(A2V,^15^N-Val2), and > 94% for Aβ1–16(A2V,^13^C(1)-Val2).

**Table 1:** Aβx–16 peptide sequences employed in this study, with labelled residues given in boldface.

Aβ1–16	DAEFRHDSGYEVHHQK-OH
Aβ3–16	EFRHDSGYEVHHQK-OH
Aβ3[pE]–16	[pE]FRHDSGYEVHHQK-OH
Aβ1[isoAsp]–16(^13^C(1)-isoAsp1) a	**D**AEFRHDSGYEVHHQK-OH
Aβ1–16(A2V,^15^N-Val2) b	D**V**EFRHDSGYEVHHQK-OH
Aβ1–16(A2V,^13^C(1)-Val2) c	D**V**EFRHDSGYEVHHQK-OH

^*a*13^C(1)-Asp/isoAsp  =  NH_2_CH(CH_2_COOH)^1**3**^ **C**OOH.

^*b*15^N-Val  =  **^15^N**H_2_CH(CHCH_3_CH_3_)COOH.

^*c*13^C(1)-Val  =  NH_2_CH(CHCH_3_CH_3_)**^13^C**OOH.

### Sample preparation

The lyophilised Aβ peptides were suspended in phosphate buffered saline (10 mM phosphate buffer, 2.7 mM KCl, 137 mM NaCl; Sigma product number P4417) at concentration of ∼1.25 mM, as determined using an extinction coefficient at 280 nm of 1280 M^–1^cm^–1^. A concentrated stock of ^65^CuCl_2_ was prepared by stirring ^65^CuO (^65^Cu, >99%; Cambridge Isotope Laboratories) in concentrated HCl and diluted in milliQ water. To the peptide solutions, 0.9 molar equivalents ^65^CuCl_2_ was added, the pH was measured using a micro-probe (Hanna Instruments, Italy) and adjusted using concentrated NaOH or HCl. Glycerol was added at 10% v/v to ensure good glass formation upon subsequent freezing. Final peptide concentrations were ∼1.0 mM. Samples were transferred to quartz EPR tubes (Wilmad, SQ-707) and snap-frozen in liquid nitrogen within minutes of metal addition.

### CW-EPR spectroscopy

X-band CW-EPR was performed using a Bruker ESP380E spectrometer fitted with a rectangular TE_102_ microwave cavity and a quartz cold finger insert. Microwave frequencies were measured with an EIP Microwave 548A frequency counter and *g* factors calibrated against the F^+^ line in CaO (*g* = 2.0001 ± 0.0002). Experimental conditions were: microwave power, 10 mW; microwave frequency, 9.42 GHz; modulation amplitude, 4 G; modulation frequency, 100 kHz; temperature, 77 K; sweep time, 168 s; time constant, 164 ms; 8 averages. Background correction was performed by subtraction of the sample-free spectrum. Second derivative spectra were obtained by differentiating the first harmonic spectrum, followed by Fourier filtering using a Hamming window to remove high frequency noise, ensuring the spectrum was not distorted. The spin Hamiltonian (SH) parameters of each coordination mode were determined from numerical simulations of the CW-EPR spectra using version 1.1.4 of the XSophe-Sophe-XeprView computer simulation software [41] on an i686 PC running Mandriva 2007, as described in detail in our earlier study [15].

### HYSCORE spectroscopy

To measure superhyperfine (shf) interactions between Cu^2+^ and remote, non-coordinating nuclei, electron spin echo envelope modulation (ESEEM) experiments were performed at X-band using a Bruker ESP380E spectrometer fitted with a Bruker ER 4118 dielectric resonator, an Oxford Instruments CF935 cryostat and a 1kW TWT amplifier. Two-dimensional hyperfine sublevel correlation (HYSCORE) experiments were carried out at 15 K using a π/2–τ–π/2–*t*_1_–π–*t*_2_–π/2–τ–echo sequence and pulse lengths of *t*_π/2_ = 16 ns and *t*_π_ = 24 ns, with a 4–step phase cycle to eliminate unwanted echoes. The time intervals *t*_1_ and *t*_2_ were varied from 48 ns to 8176 ns in steps of 64 ns (Nyquist frequency of 7.81 MHz); a value of τ = 144 ns was used to minimise blind spots below 7 MHz and to suppress ubiquitous ^1^H modulation and its subsequent frequency foldback [42]. In all spectra, the real part of the time-domain quadrature signal was selected, background corrected in both dimensions using a low-order polynomial fit, zero-filled to 256×256 data points and apodised with a Hamming window function. Following 2D-FFT, the absolute value was computed and to minimise artefacts the two-dimensional spectra were symmetrised by setting *S′*(ν*_j_*,ν*_i_*)  =  *S′*(ν*_i_*,ν*_j_*)  =  min[*S*(ν*_i_*,ν*_j_*), *S*(ν*_j_*,ν*_i_*)], where *S* and *S′* refer to the frequency-domain signal before and after symmetrisation.

## Results

### Isomerisation of Asp1 inhibits component II coordination by forming a stable 5-membered chelate

**Figure 1:**
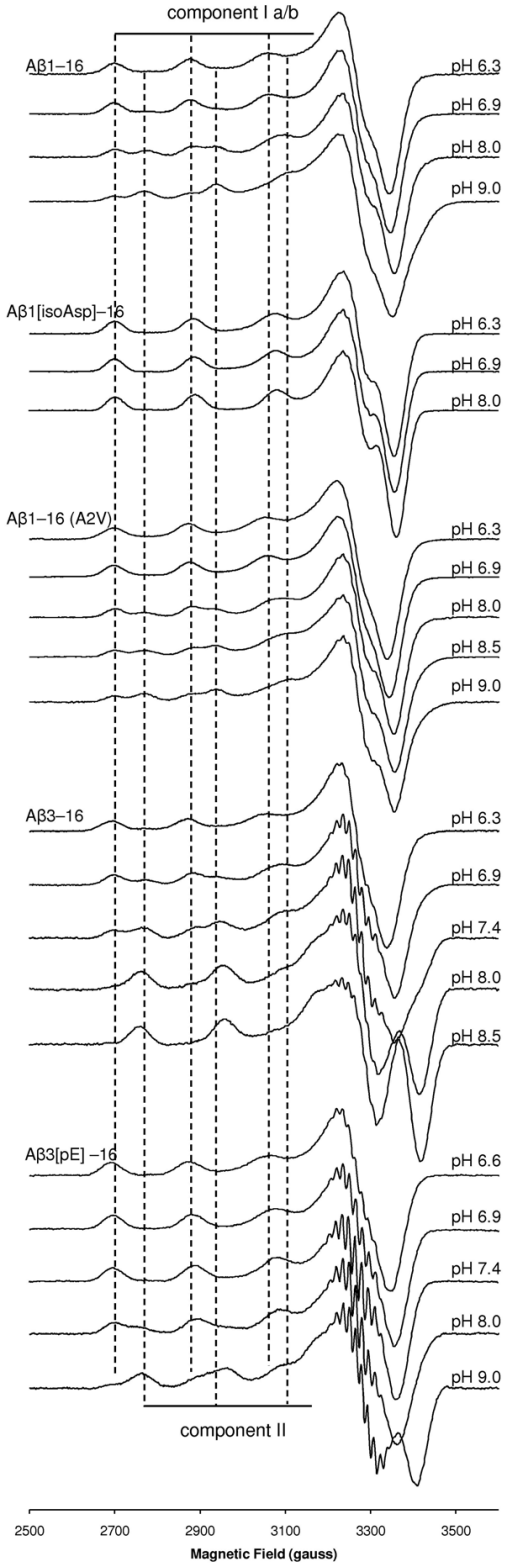
X-band (9.43 GHz) CW-EPR spectra of Cu^2+^/Aβ1–16, Cu^2+^/Aβ1–16(A2V), Cu^2+^/Aβ1[isoAsp]–16, Cu^2+^/Aβ3–16 and Cu^2+^/Aβ3[pE]–16 (0.9 equiv ^65^CuCl_2_). For comparative purposes, dashed vertical lines identify the position of the resolved *A*_||_(^65^Cu) resonances corresponding to component Ia/b and component II of Cu^2+^/Aβ1–16. Spectra of Cu^2+^/Aβ1[isoAsp]–16 and Cu^2+^/Aβ1–16(A2V) correspond to the ^13^C(1)-isoAsp1 and ^13^C(1)-Val2 labelled analogues, respectively.

**Figure 2:**
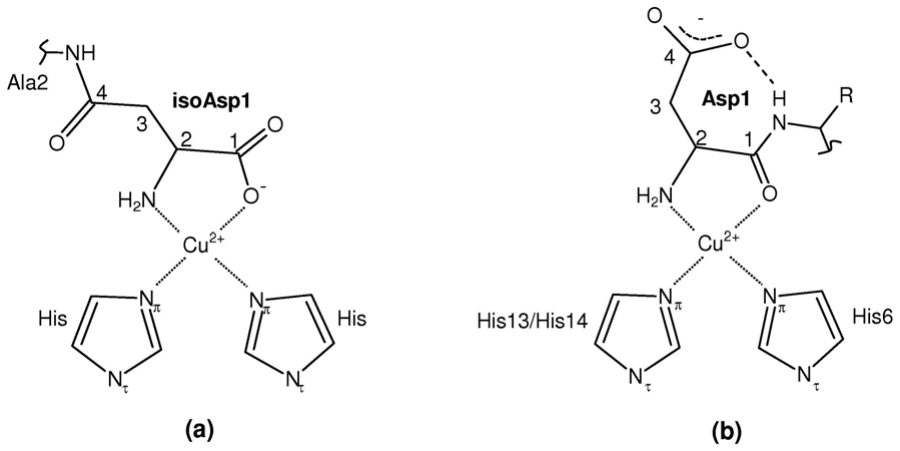
Two-dimensional representation of the 5-membered chelate formed by (a) Cu^2+^/Aβ1[isoAsp]–16 and (b) Cu^2+^/Aβ1–16 (R  =  CH_3_) and Cu^2+^/Aβ1–16(A2V) (R  =  CH_2_(CH_3_)_3_), with one possible H-bonding interaction shown. The coordination in (b) only predominates below pH 8, whereas the stable chelate in (a) remains the sole coordination mode (Figure 1).

X-band CW-EPR of Cu^2+^/Aβ1[isoAsp]–16 indicated the presence of only a single coordination mode with only subtle variation in linewidth between pH 6–8 (Figure 1), possibly due to pH-dependent structural changes beyond the first coordination sphere. This contrasts with the Aβ1–16 peptide at pH 8.0 (Figure 1), where component II is also populated, and indicates that isomerisation of Asp1 permits a highly stable coordination geometry inaccessible to the wt peptide. The SH parameters of the coordination mode are distinct from, but similar to, those of component I coordination mode of Cu^2+^/Aβ1–16 (Table 2). The above observations can be explained if Cu^2+^/Aβ1[isoAsp]–16 forms a stable 5-membered ring via the amino terminus and the carboxylate of isoAsp1 (Figure 2a), similar to the 5-membered chelate adopted by oxidised glutathione, in which the first residue of the tripeptide is isomerised glutamate [43]. We previously proposed a similar 5-membered chelate in the native peptide [16], however in this instance the oxygen coordination was via the carbonyl of Asp1 (Figure 2b).

**Table 2:** SH parameters corresponding to the different coordination modes of various Cu^2+^/Aβx–16 complexes.

Peptide	*g*_||_	*g*_⊥_	*A*_||_(^63^Cu)^a^	*A*_⊥_(^63^Cu)^a^	*a*_iso_ (ligand nuclei)	Ref
**Aβ1[isoAsp]–16**						
{NH_2_^D1^, COO^−^^D1^, N_Im_, N_Im_}	2.255±0.002	2.054±0.002	185±2	14.3±0.5	10.6±0.5	This work c
					13.1±0.5	
					14.7±0.5	
**Aβ1–16, Aβ1–16(A2V)**						
{NH_2_^D1^, CO^A2,V2^, N_Im_^H6^, N_Im_^H13/H14^}	2.272±0.005	2.056±0.005	171±3	14.5±0.5	11.3±0.5 (^14^N_a_^D1^)	[15], [16],
(“component Ia/b”)					13.0±0.5 (^14^N_Im_^H6^)	this work b,c
					14.0±0.5 (^14^N_Im_^H13/H14^)	
						
{CO^A2^, N_Im_^H6^, N_Im_^H13^, N_Im_^H14^}	2.227±0.003	2.043±0.003	157±3	21.0±1.0	15.0±1.0 (^14^N_Im_^H6^)	[15], [16],
(“component II”) d					12.5±1.0 (^14^N_Im_^H13^)	this work b,c
					12.5±1.0 (^14^N_Im_^H14^)	
**Aβ3–16, Aβ3[pE]–16**						
{3N1O} “low pH” e	2.261±0.002	2.053±0.002	183±1	16.8±0.5	12.1±0.5 (^14^N_1_)	This work b
					14.3±0.5 (^14^N_2_)	
					15.9±0.5 (^14^N_3_)	
						
{4N} “high pH” f	2.194±0.002	2.034±0.002	193±1	16.3±0.5	10.6±0.5 (^14^N_1_)	This work b
					13.2±0.5 (^14^N_2_)	
					14.2±0.5 (^14^N_3_)	
					16.1±0.5 (^14^N_4_)	
Aβ4–16						
{4N}	2.178±0.001	2.049	209±1	n.d.^g^	n.d.	[50]

^*a*^All hyperfine parameters are expressed in units of *A_i_* [10^−4^cm^−1^]  =  *A_i_* [MHz]/2.9979  =  *A*_i_ [G] × 10^4^(*g_i_β*_e_/*hc*), where *i*  =  || or ⊥, *h* is Plank's constant, *c*  =  2.9979 ×10^10^cm.s^−1^ and *β*_e_  =  9.274×10^−28^ J.G^−1^.

^*g*^To aid comparison with other work in which natural abundance copper (69% ^63^Cu, 31% ^65^Cu) has been used, hyperfine couplings have been converted from ^65^Cu to those expected for ^63^Cu using the scaling factor |*g*_n_(^65^Cu)/*g*_n_(^63^Cu)|  =  1.07. Uncertainties in parameters represent the estimated range.

^*c*^SH parameters from simulation of wt peptide [15].

^*d*^{NH_2_^D1^, N_am_^A2^, CO^A2^, N_Im_^H6^} coordination has also been proposed [17].

^*e*^Parameters based upon simulation of Cu^2+^/Aβ3[pE]–16 at pH 6.9.

^*f*^Parameters based upon simulation of Cu^2+^/Aβ3–16 at pH 8.5.

^*g*^n.d.  =  not determined.

**Figure 3:**
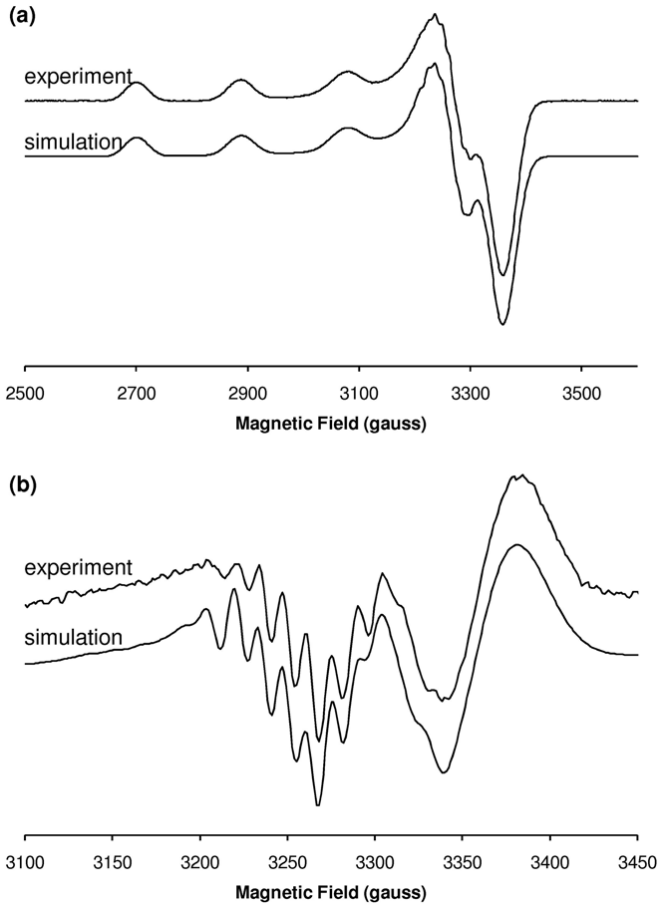
Simulation of the X-band CW-EPR spectrum of Cu^2+^/Aβ1[isoAsp]–16 at pH 6.9. (**a**) First derivative. (**b**) Second derivative, expanded around *g*_⊥_ region. Simulation parameters appear in Table 2. Experimental spectra correspond to the ^13^C(1)-isoAsp1 labelled analogue.

**Figure 4:**
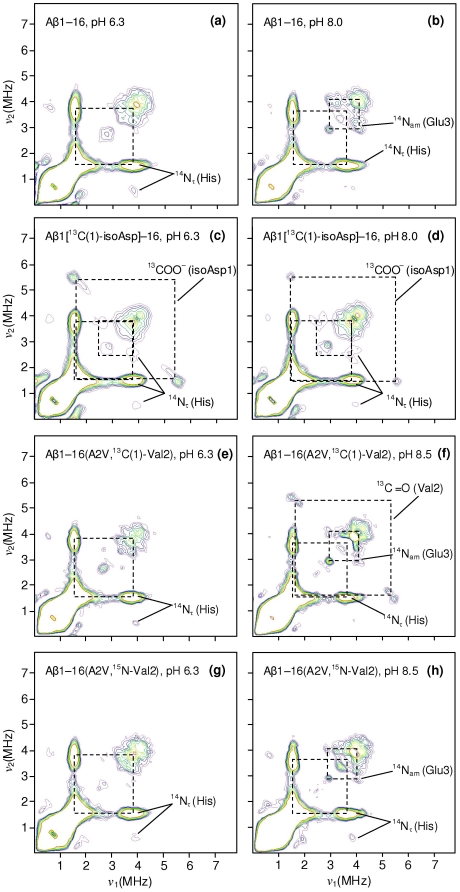
X-band (9.70 GHz) HYSCORE spectra (*τ* = 144 ns) of Cu^2+^/Aβ16 analogues (0.9 equiv ^65^CuCl_2_), obtained at 3085 G. For clarity, the cross-peaks between N_τ_ single-quantum and double-quantum transitions are not marked by dashed boxes. Grey dashed boxes highlight loss of cross-peaks.

**Figure 5:**
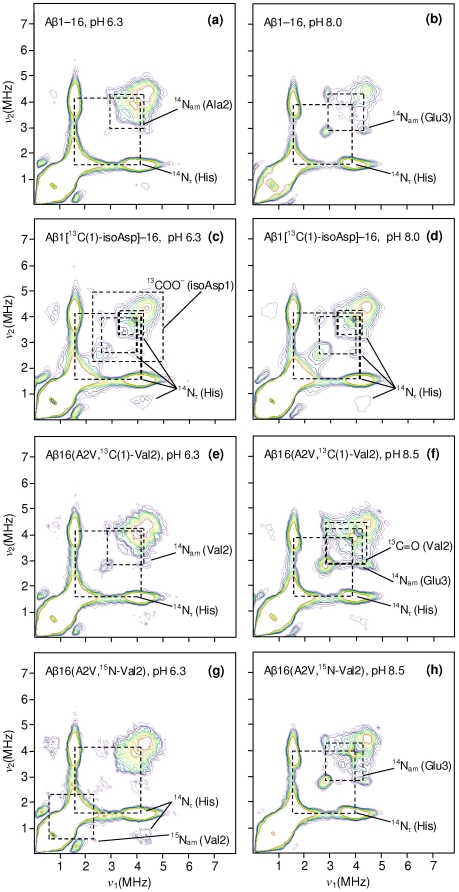
X-band (9.70 GHz) HYSCORE spectra (*τ* = 144 ns) of Cu^2+^/Aβ16 analogues (0.9 equiv ^65^CuCl_2_), obtained at 3370 G (near *g*_⊥_).

Simulation of the CW-EPR spectrum yielded principal *g* and *A*_||_(Cu) parameters consistent with a 3N1O coordination sphere [44], [45], and resonances due to metal-ligand shf coupling were also well fitted assuming 3 nitrogen ligands (Figure 3). Further evidence for the coordination shown in Figure 2a was obtained from pulsed EPR spectroscopy. Although difficult to detect near *g*_⊥_, the HYSCORE spectrum of Cu^2+^/Aβ1[^13^C(1)-isoAsp]–16 obtained at a magnetic field near *g*_||_ exhibited correlation ridges centred on the ^13^C Larmor frequency with a splitting of ∼4 MHz, consistent with equatorial carboxylate coordination of isoAsp1 (compare Figure 4c,d with Figure 5c-d). Cross peaks were also observed at (ν_dq_, ν_+_) ∼ (4, 1.6) MHz and (ν_dq_, ν_–,0_) ∼ (4, 0.8) MHz due to electron-nuclear coupling with the distal ^14^N_τ_ nucleus of equatorially coordinated His, where the ν_0_,_−_ and ν_+_ frequencies derive from transitions within the α electron spin manifold and ν_dq_ derives from the β manifold [46]. Additionally, combination peaks at (ν_dq_, ν_+_ + ν_−_) ≈ (4, 2.5) MHz and (ν_dq_, 2ν_+_) ≈ (4, 3.2) MHz were present (Figure 4c,d), similar to those observed for other Cu^2+^ binding proteins where the metal is coordinated by two His residues [47]. These combination peaks were not readily apparent in the HYSCORE spectra of unlabelled Aβ1–16 at physiological pH (Figure 4a-b, Figure 5a-b), even though we have previously established from multifrequency CW-EPR that this peptide simultaneously coordinates via at least two histidine ligands [15], [16]; however, it is frequently the case that combination lines are weak and their appearance is highly system-dependent [48]. The resonance lines from individual electron-nuclear couplings are additive in pulsed EPR methods such as HYSCORE, as opposed to the multiplicative nature of the shf interactions in CW-EPR; hence in principle the ESEEM lines of from His coordination need not originate from the same coordination mode as the lines due to carboxylate coordination. However, CW-EPR indicates only a single species is present over a wide pH range, therefore we may safely assign all of the features to the coordination proposed in Figure 2a.

### The familial A2V mutation alters the outer coordination sphere of Cu^2+^/Aβ

CW-EPR indicated the presence of an equilibrium between multiple species in Cu^2+^/Aβ1–16(A2V) very similar to wt peptide with respect to the principal *g*_||_ and *A*_||_(Cu) parameters (Table 2), as well as the position of their shf resonances (Figure S1). However, in comparison with Cu^2+^/Aβ1–16, the onset of the high-pH signal begins approximately 0.5 pH units higher as the pH is (Figure 1). The *g*_||_/*A*_||_ ratio of 142 cm for the high pH mode falls outside the normal range (105–135 cm) for normal square planar Cu^2+^ complexes, indicating that its first coordination sphere, similar to component II of Aβ1–16, is tetrahedrally distorted [44].

The similar Cu^2+^ coordination of the wt and A2V peptides was further confirmed by the appearance of ^15^N_am_(Val2) cross peaks, concomitant with the disappearance of ^14^N_am_(Val2) cross peaks, in the HYSCORE spectrum of Cu^2+^/Aβ(A2V,^15^N-Val2) at pH 6.3 (Figure 4g; Figure 5g). These features were also previously observed for Cu^2+^/Aβ1–16(^15^N^13^C-Ala2) and provide evidence for carbonyl coordination of Asp1 at low pH [16]. At pH 8.5, HYSCORE spectroscopy of Cu^2+^/Aβ1–16(A2V,^13^C(1)-Val2) identified cross peaks consistent with equatorial coordination of ^13^C = O coordination of residue 2 (Figure 4h; Figure 5h). Once again, ^13^C = O features were similarly observed for Cu^2+^/Aβ1–16(^15^N^13^C-Ala2) at pH 8.0 [16]. However, the topology of the ^13^C = O cross-peaks at both 3085 G (near *g*_||_) and 3370 G (near *g*_⊥_) is clearly different in comparison with the wt complex [16], indicating a perturbation of the outer coordination sphere of the A2V variant in coordination geometry of residue 2 upon replacing Ala with Val. Although there appear to be two pairs of ^13^C cross-peaks in Figure 4h, this clearly cannot be due to bidentate coordination of Val2 (via the amide N and carbonyl O), since only the C(1) (carbonyl) nucleus was ^13^C-labelled. There is also no evidence for an additional independent coordination mode involving C = O (Val2) that could generate a second set of cross-peaks. It is possible that the effect is the result of “holes” arising from destructive interference of the double quantum ^14^N_τ_ correlation frequency and ^13^C correlation frequency, with each possessing a different phase [49].

Overall, the combined CW-EPR and HYSCORE data indicate the identity of first coordination sphere of Cu^2+^/Aβ1–16(A2V) is the same as the wt peptide in both low pH and high pH modes; however, the replacement of the -CH_3_ side chain (Ala2) with a larger -CH(CH_3_)_2_ group (Val2) produces changes in the outer coordination sphere that lead to a modest shift in the onset of the component II-like coordination mode by approximately +0.5 pH units compared with Cu^2+^/Aβ1–16. Previous CW-EPR studies of Cu^2+^/Aβ2–16 and Cu^2+^/Aβ1–16(D1N) inferred that the ratio of components I and II of the native Cu^2+^/Aβ system is related to a hydrogen bonding interaction of COO^−^ (Asp1) with a protonated moiety in the outer coordination sphere, rather than a change of the coordinating ligands, because the SH parameters of each component appeared unchanged [50], [51]. This indirect role for COO^−^ (Asp1) as a non-coordinating ligand has since been confirmed by HYSCORE studies of Cu^2+^/Aβ1–16(^13^C(4)-Asp1), where site specific ^13^C-labelling of the side chain carboxylate failed to reveal ^13^C cross-peaks diagnostic of equatorial coordination [16]. The shift in the pH-dependent equilibrium upon replacing Ala2 with Val, without a change in SH parameters, suggests the side chain of the second residue plays a similar indirect role, however the A2V mutation shifts the equilibrium in the opposite direction (subtle decrease in high pH mode) compared with the D1N mutation (significant increase in high pH mode).

### Coordination of truncated Cu^2+^/Aβ3–16 and Cu^2+^/Aβ3[pE]–16

**Figure 6:**
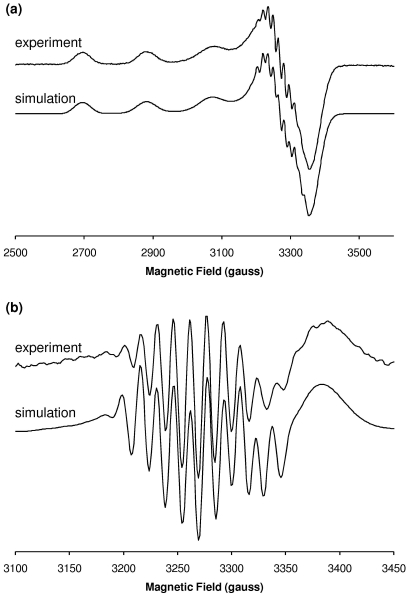
Simulation of the X-band CW-EPR spectrum of Cu^2+^/Aβ3[pE]–16 at pH 6.9. (a) First derivative. (b) Second derivative, expanded around *g*_⊥_ region. Simulation parameters appear in Table 2.

**Figure 7:**
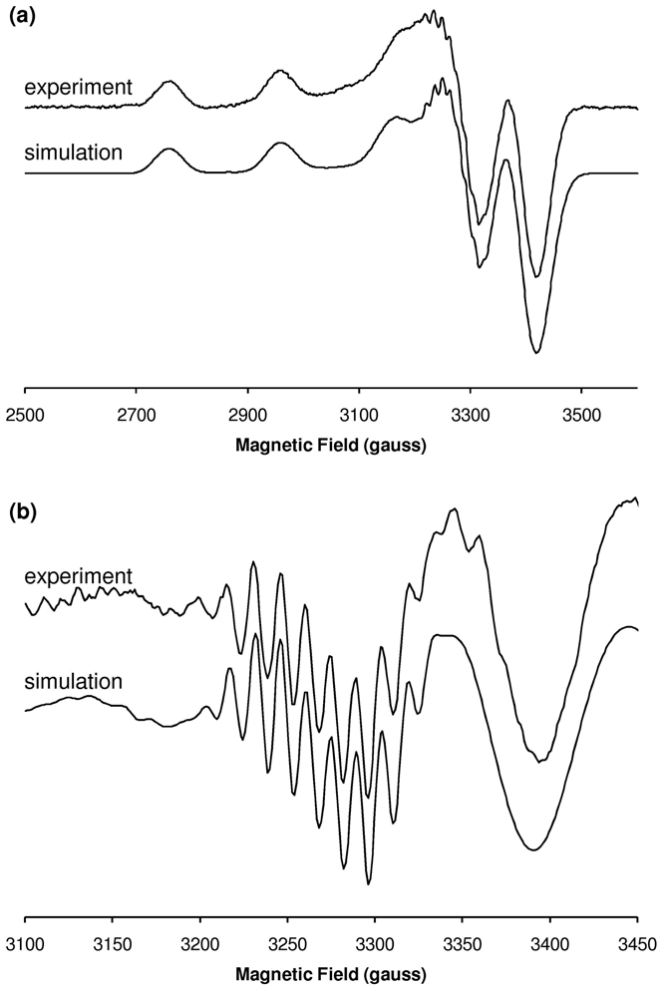
Simulation of the X-band CW-EPR spectrum of Cu^2+^/Aβ3–16 at pH 8.5. (a) First derivative. (b) Second derivative, expanded around *g*_⊥_ region. Simulation parameters appear in Table 2. Additional broadening is present in the experimental spectrum that may correspond to the onset additional 4N coordination mode(s) at higher pH or the presence of residual low pH coordination.

CW-EPR of Cu^2+^/Aβ3–16 and Cu^2+^/Aβ3[pE]–16 revealed a pH-dependent equilibrium between two main components in the physiological pH range (Figure 1). Interestingly, both Cu^2+^/Aβ3–16 and Cu^2+^/Aβ3[pE]–16 possessed very similar coordination modes, but the pH-dependence was different in each, with the onset high-pH signal beginning approximately 1 pH unit lower for Cu^2+^/Aβ3–16 (Figure S2) as the pH was raised. The positions of the *A*_||_(Cu) hyperfine resonances, as well as the metal-ligand shf resonances, were highly similar for Cu^2+^/Aβ3–16 and Cu^2+^/Aβ3[pE]–16 at both low and high pH, suggesting that neither the free amino terminus nor the COO^−^ (Glu3) side chain of Aβ3–16 directly coordinates Cu^2+^. The principal *g* and *A*_||_(Cu) parameters obtained from numerical simulations (Figure 6, Figure 7) were consistent with a 3N1O coordination mode at low pH and a 4N mode at high pH [44], [45] and these assignments were further supported by the simulation of the shf resonances (Table 2). HYSCORE spectroscopy of Cu^2+^/Aβ3–16 and Cu^2+^/Aβ3[pE]–16 each showed similar features due to histidine ^14^N_τ_ nuclei at pH <7 and pH 9.0 (Figure S3), indicating that at least one His side chain coordinates in both the 3N1O and 4N modes, with the remaining nitrogen ligands coming from deprotonated backbone amide groups. The absence of ^14^N features characteristic of a non-coordinating nearby amide N (Figure S3) indicated a carbonyl can be excluded as an oxygen ligand in the low pH 3N1O coordination mode.

Raman spectroscopy and aggregation studies of Cu^2+^ coordination of related Aβ3–9(E3A), Aβ3–9(H6A), Aβ3–9(D7A) or Ac-Aβ3–9 peptides suggested that the amino terminus, His6 and the carboxylate groups of Glu3 and Asp7 coordinate Cu^2+^ in Aβ3–9 at pH 6 [52]. The coordination of the amino group was suggested based upon the ability of Cu^2+^/Aβ3–9 to form amyloid between pH 4–8, but not Cu^2+^/Ac-Aβ3–9 [52]. However, it is unclear whether simultaneous coordination of each of the above mentioned residues was implied; certainly, the simultaneous coordination of the amino terminus and the carboxylate side chain of Glu3 would require a highly unfavourable 7-membered chelate ring. Direct comparisons between Cu^2+^/Aβ3–9 and Cu^2+^/Aβ3–16 are difficult to make, since no EPR data is available for Aβ3–9 and the consequences for Cu^2+^ coordination of truncation at Gly9 on are unknown.

A different pH-dependence of the occupancy of the 3N1O and 4N coordination modes for Cu^2+^/Aβ3–16 and Cu^2+^/Aβ3[pE]–16, without a major perturbation of their SH parameters, suggests the N-terminal Glu plays an indirect role in a manner similar to that of Asp1 in controlling the ratio of components I and II signals of Cu^2+^/Aβ1–16 and Cu^2+^/Aβ1–16(D1N). A key difference, however, is that the loss of the carboxylate side chain upon cyclisation of Glu3 leads to a reduction of the occupancy of the high-pH species, which compares with the increase in the high pH species observed for Cu^2+^/Aβ1–16(D1N) and Cu^2+^/Aβ2–16 in comparison with Cu^2+^/Aβ1–16 [50].

## Discussion

In this study, we have investigated the Cu^2+^coordination of four model Aβ peptides with physiologically relevant N-terminal modifications or truncations. With the exception of Cu^2+^/Aβ1[isoAsp]–16, which possesses a single dominant coordination mode, all peptides exhibited equilibria between multiple pH-dependent Cu^2+^ coordination modes in the physiological pH range.

The CW-EPR spectra of Cu^2+^/Aβ1[isoAsp]–16 showed that the shorter carboxylate-bearing side chain of isoAsp1 enables a stable five-membered ring to form between Cu^2+^, the amino terminus and the carboxylate oxygen. HYSCORE spectroscopy provided direct confirmation of equatorial coordination by the carboxylate oxygen of isoAsp1, in addition to two His side chains. The {NH_2_^D1^, COO^−^^D1^, 2N_Im_} coordination is similar to the 5-membered ring we previously proposed for wt Aβ at low pH, involving the amino nitrogen and the backbone carbonyl of Asp1 (components Ia and Ib) [16]; however, its stability is greatly increased compared with component I such that the high pH coordination mode observed at pH >7 in wt Aβ1–16 (component II), or any potential alternative arising from the modified peptide backbone, is eliminated at all physiologically relevant pH values.

The Aβ(A2V) peptide derives from a recently identified familial APP mutation [39]. A number of observations in this study indicated that the A2V mutation alters the outer coordination sphere as compared with Cu^2+^/Aβ1–16, but not the identity of the coordinating ligands. Comparison of CW-EPR spectra of Cu^2+^/Aβ1–16(A2V) identified the presence of low pH and high pH signals with principal *g* and *A* values and ligand shf splitting almost indistinguishable from components Ia/b and component II of Cu^2+^/Aβ1–16. HYSCORE spectroscopy confirmed that C = O (Val2) coordinates in the high pH mode in a manner similar to Ala2 in Cu^2+^/Aβ1–16 [16], and that C = O (Asp1) coordination occurs at low pH analogous to Cu^2+^/Aβ1–16 [16]. Noteworthy was the subtle shift in the pH dependence of the coordination modes by ∼0.5 pH units in response to the A2V mutation. Coupled with the known pH dependence of the coordination modes on the outer sphere interactions of COO^−^ (Asp1) [51], this suggests the mutation may either provide steric influences that could either enhance the strength of any H-bonding or salt bridge [53] interactions involving Asp1, or destabilise the high pH coordination mode via its side chain interactions independently of Asp1.

In the case of Cu^2+^/Aβ3–16 and Cu^2+^/Aβ3[pE]–16, CW-EPR suggested that the N-terminus plays a key role in controlling the ratio of the low and high pH signals without directly coordinating Cu^2+^. Since changes in the first coordination sphere do not always lead to significant perturbation of the SH parameters, it remains possible that the coordinating ligands in one or both modes are in fact different for Cu^2+^/Aβ3–16 and Cu^2+^/Aβ3[pE]–16, in particular that Glu3 coordinates Cu^2+^/Aβ3–16. However, the shift in the pH dependence may also be rationalised by differences in outer coordination sphere interactions alone. Since N-terminal pGlu is known to affect a protein's structural stability [54], our observations suggest that pGlu could alter the relative stability of the high pH mode in Cu^2+^/Aβ3[pE]–16, either by participating hydrogen bonding interactions in the outer coordination sphere (eg. as an H-bond acceptor via the O_ε_ of pGlu and/or an H-bond donor via its amide N) [54], by eliminating H-bonding or salt bridge interactions [53] that are present in Cu^2+^/Aβ3–16, or by increasing the pK_a_ of a Cu^2+^ ligand that directly participates in the high pH mode (eg. between the O_ε_ of pGlu and a coordinating backbone amide N). These possibilities, together with more definitive ligand assignments, including the identity of the coordinating oxygen, imidazole and amide nitrogen ligands, await quantitative assessment by isotopic labelling of residue 3 and other key residues. However, it is clear from the present data that cyclisation of Glu3 has a significant impact on the Cu^2+^ coordination properties Aβ with N-terminal pyroglutamate.

### Could Cu^2+^ promote N-terminal truncation of Aβ?

While cyclisation of the N-terminal glutamate of Aβ3–x appears to be enhanced by glutaminyl cyclase [55], [56], the process leading to the production of the Aβ3–x precursor itself remains unclear. Recently, the extracellular glutamyl aminopeptidase (aminopeptidase A) has been shown to cleave Asp1 from Aβ in both cell free and cell culture models [57], however this alone appears insufficient to explain the generation of truncations at position 3. In principle, a second successive degradation step on Aβ2–x by alanyl aminopeptidase (aminopeptidase N), which accounts for approximately 80% of the total soluble aminopeptidase activity in the human cortex [58], [59], could account for Aβ3–x formation in conjunction with glutamyl aminopeptidase activity. However, alanyl aminopeptidase has limited activity against Ala-X sequences with acidic X residues [58], aminopeptidase and didpetidyl aminopeptidase activity is not significantly higher in soluble extracts of frontal cortex from AD brains [59], and overall Aβ production does not increase most in familial forms of AD or with age [40]. Hence, there is presently insufficient evidence to conclude that any specific aminopeptidase activity is responsible for generation of the Aβ3–x precursor of Aβ3[pE]–x in AD.

Kowalik-Jankowska and co-workers analysed the products of Cu^2+^ catalyzed oxidation of human and mouse (R5G, Y10F, H13R) Aβ1–16 in the presence of hydrogen peroxide [60], [61]. A range of oxidatively modified species including fragmentation products obtained by peptide bond cleavage were observed. For the mouse peptide, Aβ3–16 fragments generated by α-amidation and diamide pathways were identified (the latter fragment also contained 2-oxo-His). Since Cu^2+^/Aβ interactions are believed to produce hydrogen peroxide *in vivo* [62], [63] and the Aβ3–16 fragment generated by the diamide pathway leaves a free amino nitrogen, this represents a plausible source of truncated Aβ3–16. However, while a number of fragmentation products were generated in both mouse and human peptides, specific Aβ3–16 products were reported only for the mouse Aβ [60].

**Figure 8:**
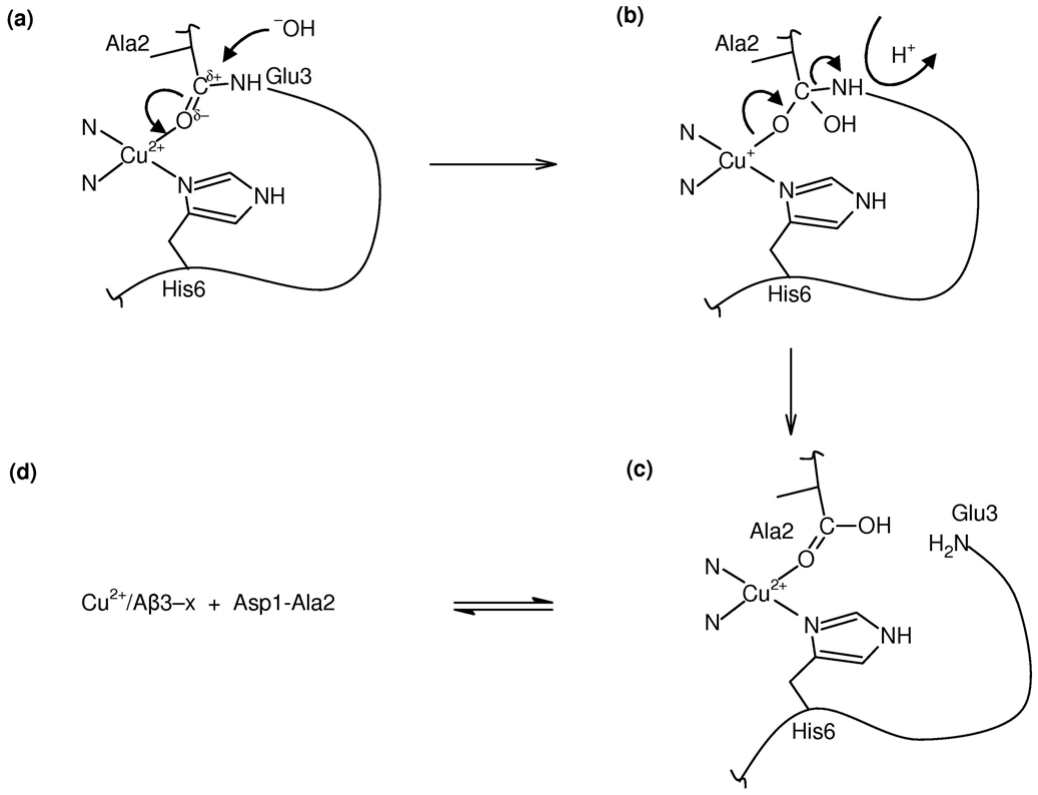
Postulated mechanism of Cu^2+^-promoted amide hydrolysis leading to Aβ truncation at Glu3. (a) Coordination of Ala2 (component II coordination mode) polarises the carbonyl carbon, allowing nucleophilic attack by OH^−^, leading to (b) the formation of a tetrahedral intermediate (TI), possibly via a Cu^+^ oxidation state (alternatively, the coordination may be Cu^2+^–O^−^–C–); (c) subsequent breakdown of the TI involving cleavage of the amide bond and protonation of the leaving amide. This latter step may involve the participation of a nearby amino acid side chain for proton transfer to the leaving amide nitrogen. Additional transient interactions with other cofactors *in vivo* could be required to promote formation, and importantly the breakdown, of the TI. Other biological nucleophiles may also be considered in step (a), such thiols (eg. glutathione, L-homocysteine) or a serine hydroxyl group. The geometry of the coordinating ligands is drawn schematically only.

Upon identification of C = O (Ala2) as the oxygen ligand in component II coordination of Aβ, we recently proposed an alternate peptide cleavage mechanism based upon Cu^2+^-promoted amide hydrolysis, following polarisation of the C = O bond of the coordinating carbonyl of Ala2 [16]. Such a process is proposed to underlie the activity of metallohydrolases such as carboxypeptidase A and thermolysin, and possible mechanisms have been described in some detail [64]–[66]. Based upon such mechanisms, a hypothetical hydrolysis reaction generating Aβ3–16 is presented in Figure 8. This mechanism could involve a Cu^+^ intermediate (Figure 8), allowing for the additional possibility of oxygen activation and ROS formation as a side reaction.

Unlike metallohydrolases, the requirement for intramolecular cleavage of the amide bond in Aβ, as opposed to enzymatic cleavage of an extramolecular substrate, naturally precludes a catalytic mechanism. While the Aβ1–x stabilised in senile plaques contains bound Cu^2+^ [67] and could conceivably undergo hydrolysis, *soluble* Aβ3[pE]–x species that are thought to be involved in disease initiation must be generated prior to formation of extracellular amyloid and hence prior to Aβ turnover. While very large rate increases have been reported in the presence of metals such as copper and zinc, amide hydrolysis generally involves a tetrahedral intermediate with a poor RNH- leaving group that must be protonated either prior to or in concert with C-N cleavage, meaning the reaction is usually very slow at pH 7 (Figure 8) [64]. Ie. the number of cleavage events that would occur prior to turnover of soluble Aβ1–x would be exceedingly small. Nevertheless, the very gradual increase in levels of N-terminally truncated Aβ as disease progresses implies that amide hydrolysis occurs infrequently and as such only an exceedingly small rate constant would be required.

The rate of amide hydrolysis, in which the limiting step is believed to be breakdown of the tetrahedral intermediate [64], may depend critically upon stereoelectronic constraints of the metal and the carbonyl ligand [64], [68], a nearby amino acid side chain for proton transfer to the leaving amide nitrogen [66], as well as the identity of the attacking nucleophile. Hence, post-translational modifications, familial mutations, binding partner or receptor interactions *in vivo*, or formation low molecular weight oligomeric species may modulate such a process. The appearance of Aβ1(isoAsp)–x species in AD plaques is consistent with a hydrolysis mechanism involving C = O(Ala2), since coordination of C = O(Ala2) is absent in Cu^2+^/Aβ1[isoAsp]–16. In the case of the familial A2V mutant, the subtle alteration in the pH dependence and the change in the carbonyl coordination geometry might be expected to affect the rate of hydrolysis. CHO and COS-7 cells transfected with the A673V mutation have increased secretion of Aβ11–40, Aβ11–42 and Aβ3[pE]–42 compared with controls transfected with wt APP; however, within the limits of uncertainty, levels of Aβ1–40 and Aβ1–42 were also increased by the same ratio [39]. *In vivo* data on Aβ3[pE]–x levels for this inherited form of AD are presently unknown. Verification of the mechanism *in vitro* may be complicated by the requirement for additional cofactors present *in vivo* and a potentially very low rate constant. Further investigation of the *C. elegans* model, which predominantly expresses Aβ3–42 [31], may help to elucidate the *in vivo* mechanism of N-terminal truncation.

In conclusion, we have examined the changes in Cu^2+^ coordination associated with N-terminal modifications that accompany the accumulation of extracellular Aβ *in vivo*, including isomerisation, truncation and cyclisation. Using CW and pulsed EPR spectroscopy, we have examined the changes in Cu^2+^ coordination that accompany these physiologically relevant N-terminal modifications to the Aβ1–16 peptide. Isomerisation of Asp1 enables Cu^2+^/Aβ1[isoAsp]–16 to form a 5-membered ring via the amino terminus and the carboxylate of isoAsp1, with the remaining ligands being supplied by His side chains. This coordination is similar to component I of native Aβ, except the latter forms a less stable 5-membered chelate involving the amino terminus and the carbonyl of Asp1. The stability of the Cu^2+^/Aβ1[isoAsp]–16 coordination ensures that it is the only species observed in CW-EPR spectra in the physiological pH range. The recently identified familial A2V mutation appears to preserve the first Cu^2+^ coordination sphere adopted by Cu^2+^/Aβ1–16. However, changes in outer coordination sphere interactions lead to a modest decrease in the relative occupancy of the low and high pH modes. Examination of the Cu^2+^ coordination of truncated Aβ3–16 and cyclised Aβ3[pE]–16 revealed an equilibrium between a 3N1O species at low pH and a 4N species at high pH. The similarity of the two modes suggests that the identity of the Cu^2+^ coordinating ligands in Cu^2+^/Aβ3–x peptide and Cu^2+^/Aβ3[pE]–x are the same; however, the pH dependence is dramatically different for each peptide, with cyclisation of the negatively-charged carboxylate of Glu3 leading to a reduction in the relative occupancy of the 4N coordination mode. N-terminal pyroglutamate Aβ peptides are reported to be more toxic than their non-truncated counterparts [28], [29], appear to accumulate as disease progresses [30] and form the major component of PIB-positive amyloid observed in the AD brain by PET imaging [26]. While the origin of these truncated species *in vivo* remains unclear, we speculate that Cu^2+^-promoted amide hydrolysis may provide a possible mechanism.

## Supporting Information

Figure S1 **Comparison of X-band CW‐EPR spectra of Cu^2+^/Aβ1–16 and Cu^2+^/Aβ1–16(A2V).** Spectra of Cu^2+^/Aβ1–16 were acquired in PBS adjusted to (**a**) pH 6.9, (**b**) pH 8.0 (**c**) pH 9.0. (**d**) Weighted subtraction of spectrum *a* from spectrum *b* to isolate component II. (**e**) Weighted subtraction of spectrum *a* from spectrum *c* showing additional broadening in the *g*_⊥_ region, due to spectral “contamination” arising from partial population of an additional high pH (4N) coordination mode. (**f**) Spectrum of Aβ1–16 at pH 10.6. Although this pH was used in ref [17] to demonstrate a change in shf structure of Cu^2+^/Aβ1–16(^15^N‐Ala2) as evidence of N_am_^A2^ coordination in component II, the full spectrum, and hence the shf pattern, at this pH clearly corresponds to a different 4N coordination mode. A comparison of the second derivative spectra in PBS 6.9 of (**g**) Aβ1–16 (**h**) Aβ1–16(^15^N^13^C‐Ala2) (**i**) Aβ1–16(A2V,^13^C(1)‐Val2) and (**j**) Aβ1–16(A2V,^15^N-Val2), shows the position of the shf resonances of Cu^2+^/Aβ1–16(A2V) are very similar to the wt complex in component I coordination. The lower spectral resolution of the A2V complex may reflect a greater propensity to aggregate [39]. Broadening of the shf resonances is seen in spectrum *g* compared with *f*, arising from unresolved ^15^N_am_^A2^ interactions associated with C=O^D1^ coordination due to ^15^N-labelling of Ala2. For component II‐type coordination, comparison of second derivative spectra of (**k**) Cu^2+^/Aβ1–16, pH 8.0 – pH 6.9 (**l**) Cu^2+^/Aβ1–16(^15^N^13^C‐Ala2), pH 8.0 – pH 6.9 (**m**) Cu^2+^/Aβ1–16(A2V,^13^C‐Val2), pH 8.5 – pH 6.9 and (**n**) Cu^2+^/Aβ1–16(A2V,^15^N‐Val2), pH 8.5 – pH 6.9, shows that the positions of the shf resonances of Cu^2+^/Aβ1–16(A2V) are similar to the wt complex, but slightly perturbed; this is consistent with the different ^13^C=O^A2^ correlation ridges observed in the HYSCORE spectra of Cu^2+^/Aβ1–16(^13^C‐Val2) at pH 8.5. Broadening of the shf resonances is seen in spectrum *l* compared with *k*, arising from unresolved ^13^C shf interactions associated with C=O^A2^ coordination due to uniform ^13^C‐labelling of Ala2. Dashed vertical lines in spectra *g* – *n* represent the approximate position of the shf resonances of Cu^2+^/Aβ1–16 for comparative purposes. The Aβ1–16(^15^N^13^C‐Ala2) peptide was prepared as described previously [16].
(TIF)

Figure S2 **Comparison of low and high‐pH Cu^2+^ coordination modes from X‐band CW‐EPR spectra of N‐terminally truncated Aβ.** Both coordination modes are highly similar for each peptide; however, the onset of the high‐pH signal begins approximately 1 pH unit lower for Cu^2+^/Aβ3–16 as the pH is raised. Dashed vertical lines identify the approximate position of the resolved *A*_||_(^65^Cu) resonances of the low and high pH modes.
(TIF)

Figure S3 **X‐band HYSCORE spectra (*τ*=144 ns) of Cu^2+^/Aβ3–16 and Cu^2+^/Aβ3[pE]–16 analogues (0.9 equiv ^65^CuCl_2_), obtained at 3150 G and 3370 G (near *g*_⊥_).** Spectrum in (g) was acquired with a smaller number of data points in the time domain compared with the rest of the data set.
(TIF)
