# Correction: Alzheimer's Aβ Peptides with Disease-Associated N-Terminal Modifications: Influence of Isomerisation, Truncation and Mutation on Cu^2+^ Coordination

**DOI:** 10.1371/journal.pone.0102192

**Published:** 2014-07-01

**Authors:** The PLOS ONE Staff

There are errors in Table 2. Please see the corrected Table 2 below.

**Table:** **Table 2** **.** SH parameters corresponding to the different coordination modes of various Cu^2+^/Aβx–16 complexes.

Peptide	*g*_||_	*g*_⊥_	*A*_||_(^63^Cu)^a^	*A*_⊥_(^63^Cu)^a^	*a*_iso_ (ligand nuclei)	Ref
**Aβ1[isoAsp]–16**						
{NH_2_^D1^, COO^–^^D1^, N_Im_, N_Im_}	2.255 ± 0.002	2.054 ± 0.002	185 ± 2	14.3 ± 0.5	10.6 ± 0.5 (^14^NH_2_^D1^)	This work *^c^*
					13.1 ± 0.5 (^14^N­_Im_)	
					14.7 ± 0.5 (^14^N­_Im_)	
**Aβ1–16, Aβ1–16(A2V)**						
{NH_2_^D1^, CO^D1^, N_Im_^H6^, N_Im_^H13/H14^}	2.272 ± 0.005	2.056 ± 0.005	171 ± 3	14.5 ± 0.5	11.3 ± 0.5 (^14^NH_2_^D1^)	[15,16],
(“component Ia/b”)					13.0 ± 0.5 (^14^N­_Im_^H6^)	this work *^b,c^*
					14.0 ± 0.5 (^14^N_Im_^H13/H14^)	
{CO^A2,V2^, N_Im_^H6^, N_Im_^H13^, N_Im_^H14^}	2.227 ± 0.003	2.043 ± 0.003	157 ± 3	21.0 ± 1.0	15.0 ± 1.0 (^14^N­_Im_^H6^)	[15,16],
(“component II”) *^d^*					12.5 ± 1.0 (^14^N­_Im_^H13^)	this work *^b,c^*
					12.5 ± 1.0 (^14^N­_Im_^H14^)	
**Aβ3–16, Aβ3[pE]–16**						
{3N1O} “low pH” *^e^*	2.261 ± 0.002	2.053 ± 0.002	183 ± 1	16.8 ± 0.5	12.1 ± 0.5 (^14^N_1_)	This work *^b^*
					14.3 ± 0.5 (^14^N_2_)	
					15.9 ± 0.5 (^14^N_3_)	
{4N} “high pH” *^f^*	2.194 ± 0.002	2.034 ± 0.002	193 ± 1	16.3 ± 0.5	10.6 ± 0.5 (^14^N_1_)	This work *^b^*
					13.2 ± 0.5 (^14^N_2_)	
					14.2 ± 0.5 (^14^N_3_)	
					16.1 ± 0.5 (^14^N_4_)	
**Aβ4–16**						
{4N}	2.178 ± 0.001	2.049	209 ± 1	n.d.*^g^*	n.d.	[50]

a All hyperfine parameters are expressed in units of *A_i_* [10^−4^cm^−1^]  =  *A_i_* [MHz] / 2.9979  =  *A*_i_ [G] × 10^4^(*g_i_β*_e_/*hc*), where *i*  =  || or ⊥, *h* is Plank’s constant, *c*  =  2.9979 ×10^10^cm.s^−1^ and *β*_e_  =  9.274 × 10^−28^ J.G^−1^. *^b^* To aid comparison with other work in which natural abundance copper (69% ^63^Cu, 31% ^65^Cu) has been used, hyperfine couplings have been converted from ^65^Cu to those expected for ^63^Cu using the scaling factor |*g*_n_(^65^Cu) / *g*_n_(^63^Cu)|  =  1.07. Uncertainties in parameters represent the estimated range. *^c^* SH parameters from simulation of wt peptide [15]. *^d^* {NH_2_^D1^, N_am_^A2^, CO^A2^, N_Im_^H6^} coordination has also been proposed [17]. *^e^* Parameters based upon simulation of Cu^2+^/Aβ3[pE]–16 at pH 6.9. *^f^* Parameters based upon simulation of Cu^2+^/Aβ3–16 at pH 8.5. *^g^* n.d.  =  not determined.
