# Supplementary figures and images for: Alzheimer's Aβ Peptides with Disease-Associated N-Terminal Modifications: Influence of Isomerisation, Truncation and Mutation on Cu2+ Coordination
Source: PLoS One. 2010 Dec 30;5(12):e15875. doi: 10.1371/journal.pone.0015875 (PMC3012727; doi:10.1371/journal.pone.0015875)

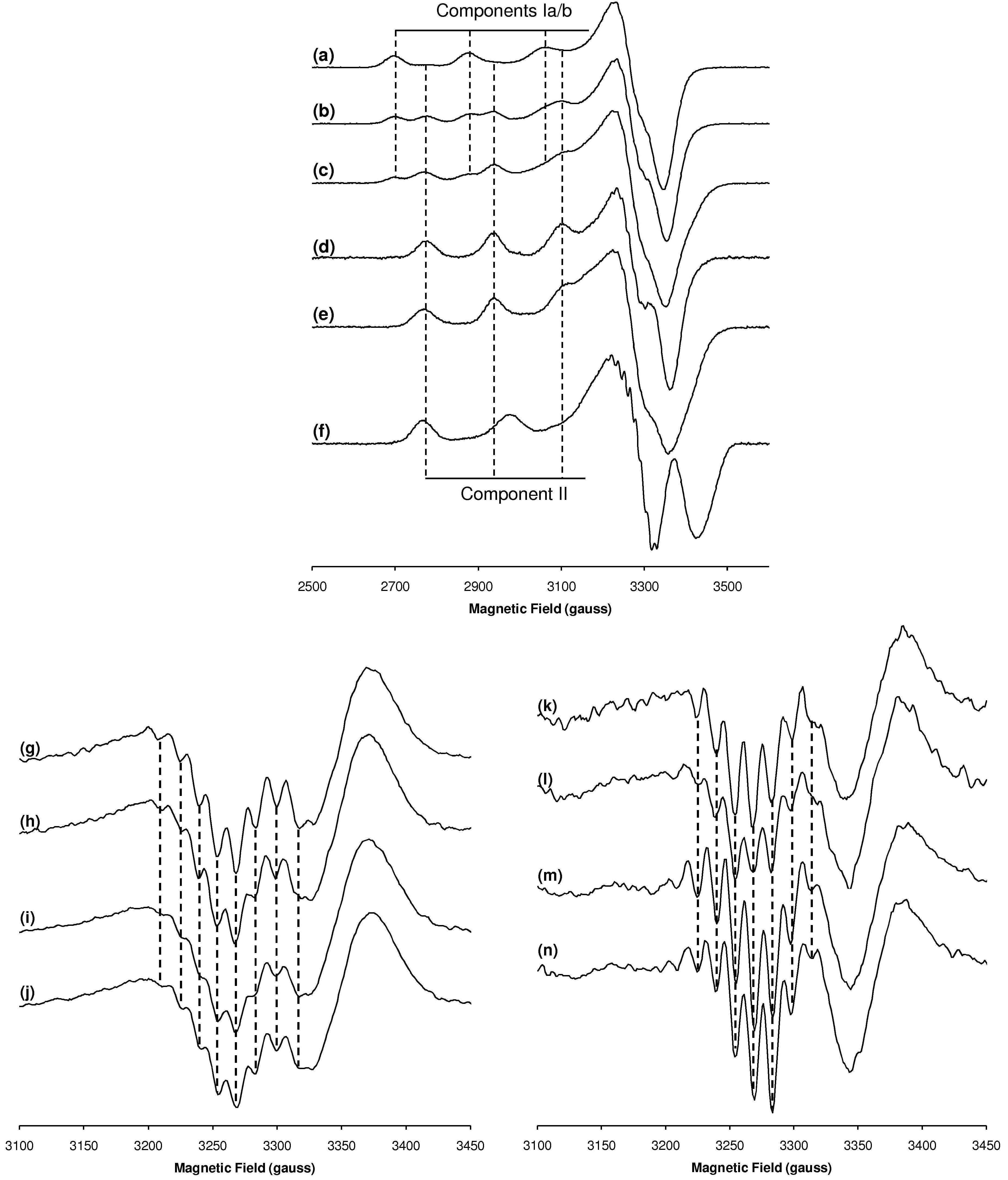

Supplement: Figure S1 — Comparison of X-band CW‐EPR spectra of Cu2+/Aβ1–16 and Cu2+/Aβ1–16(A2V). Spectra of Cu2+/Aβ1–16 were acquired in PBS adjusted to (a) pH 6.9, (b) pH 8.0 (c) pH 9.0. (d) Weighted subtraction of spectrum a from spectrum b to isolate component II. (e) Weighted subtraction of spectrum a from spectrum c showing additional broadening in the g ⊥ region, due to spectral “contamination” arising from partial population of an additional high pH (4N) coordination mode. (f) Spectrum of Aβ1–16 at pH 10.6. Although this pH was used in ref [17] to demonstrate a change in shf structure of Cu2+/Aβ1–16(15N‐Ala2) as evidence of Nam A2 coordination in component II, the full spectrum, and hence the shf pattern, at this pH clearly corresponds to a different 4N coordination mode. A comparison of the second derivative spectra in PBS 6.9 of (g) Aβ1–16 (h) Aβ1–16(15N13C‐Ala2) (i) Aβ1–16(A2V,13C(1)‐Val2) and (j) Aβ1–16(A2V,15N-Val2), shows the position of the shf resonances of Cu2+/Aβ1–16(A2V) are very similar to the wt complex in component I coordination. The lower spectral resolution of the A2V complex may reflect a greater propensity to aggregate [39]. Broadening of the shf resonances is seen in spectrum g compared with f, arising from unresolved 15Nam A2 interactions associated with C=OD1 coordination due to 15N-labelling of Ala2. For component II‐type coordination, comparison of second derivative spectra of (k) Cu2+/Aβ1–16, pH 8.0 – pH 6.9 (l) Cu2+/Aβ1–16(15N13C‐Ala2), pH 8.0 – pH 6.9 (m) Cu2+/Aβ1–16(A2V,13C‐Val2), pH 8.5 – pH 6.9 and (n) Cu2+/Aβ1–16(A2V,15N‐Val2), pH 8.5 – pH 6.9, shows that the positions of the shf resonances of Cu2+/Aβ1–16(A2V) are similar to the wt complex, but slightly perturbed; this is consistent with the different 13C=OA2 correlation ridges observed in the HYSCORE spectra of Cu2+/Aβ1–16(13C‐Val2) at pH 8.5. Broadening of the shf resonances is seen in spectrum l compared with k, arising from unresolved 13C shf interactions associated with C=OA2 coordination due to u [file pone.0015875.s001.tif]

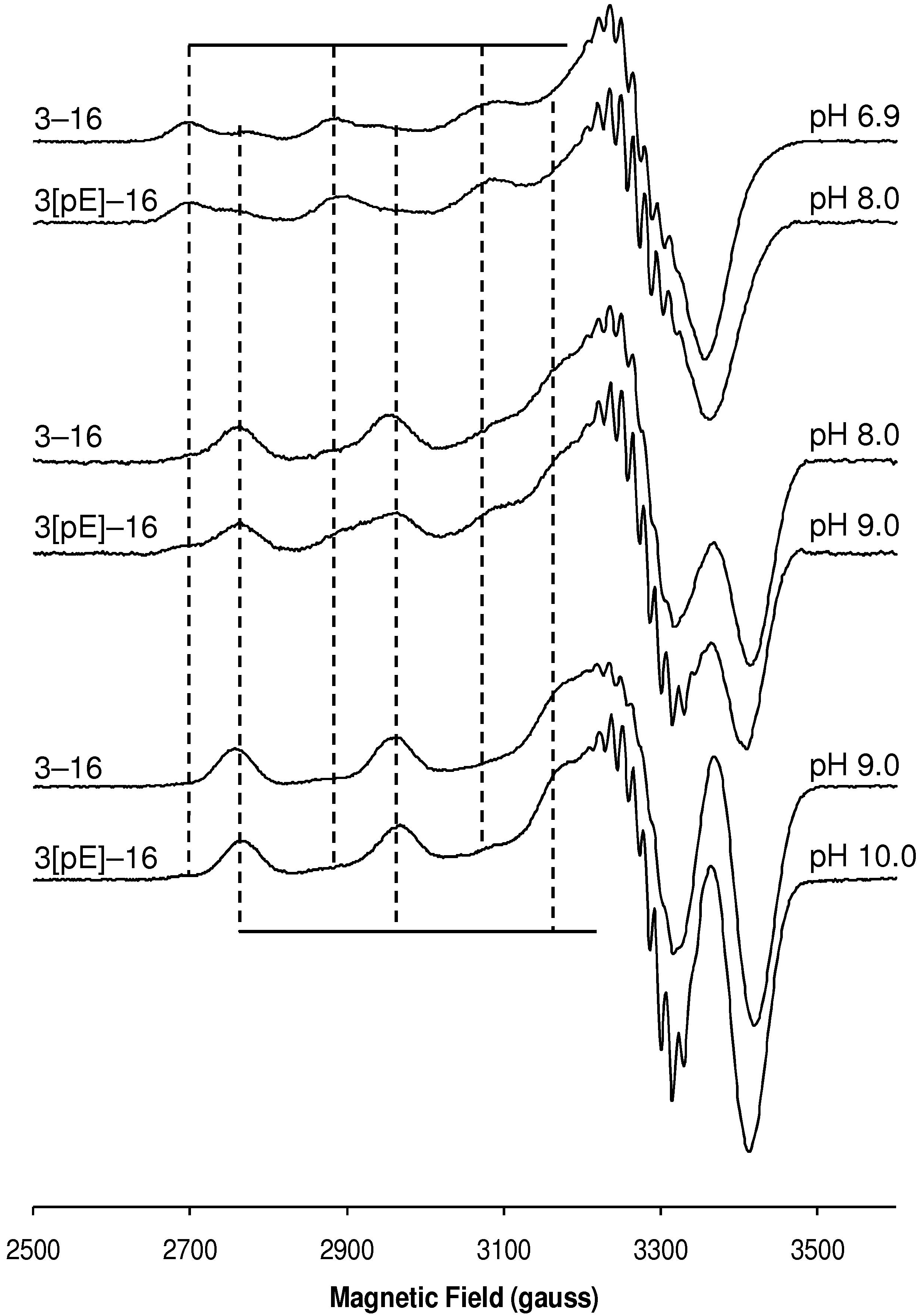

Supplement: Figure S2 — Comparison of low and high‐pH Cu2+ coordination modes from X‐band CW‐EPR spectra of N‐terminally truncated Aβ. Both coordination modes are highly similar for each peptide; however, the onset of the high‐pH signal begins approximately 1 pH unit lower for Cu2+/Aβ3–16 as the pH is raised. Dashed vertical lines identify the approximate position of the resolved A ||(65Cu) resonances of the low and high pH modes. (TIF) [file pone.0015875.s002.tif]

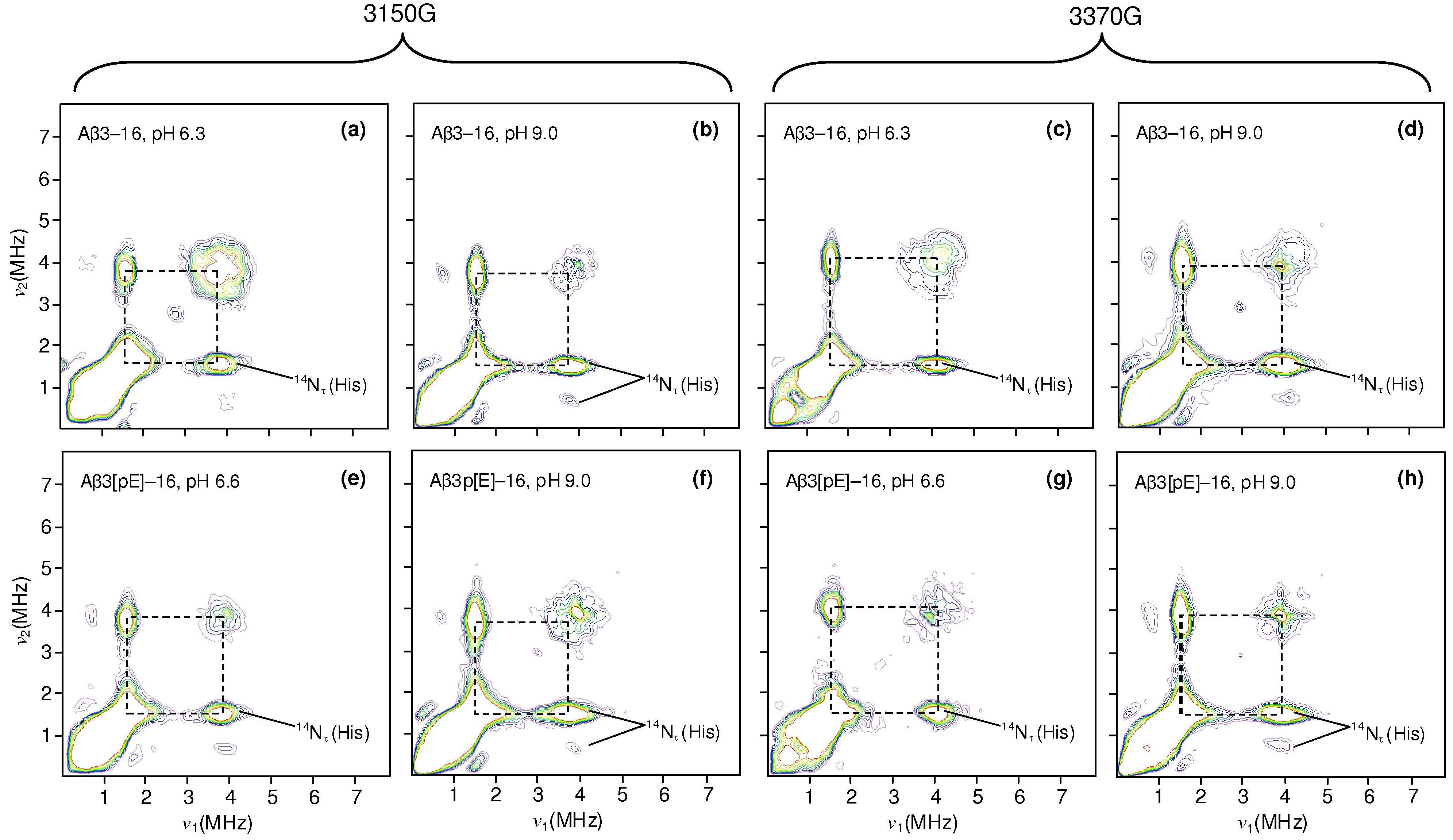

Supplement: Figure S3 — X‐band HYSCORE spectra (τ=144 ns) of Cu2+/Aβ3–16 and Cu2+/Aβ3[pE]–16 analogues (0.9 equiv 65CuCl2), obtained at 3150 G and 3370 G (near g⊥). Spectrum in (g) was acquired with a smaller number of data points in the time domain compared with the rest of the data set. (TIF) [file pone.0015875.s003.tif]
